# Target Mapping in Cancer: Ligandable Protein Pockets on 3D OncoPPI Networks

**DOI:** 10.3390/ph18070958

**Published:** 2025-06-25

**Authors:** Daniela Trisciuzzi, Orazio Nicolotti, Gabriele Cruciani, Gabriele Menna, Lydia Siragusa

**Affiliations:** 1 Department of Pharmacy, Pharmaceutical Sciences, Università Degli Studi di Bari “Aldo Moro”, Via E. Orabona, 4, 70125 Bari, Italy; daniela.trisciuzzi@uniba.it (D.T.); orazio.nicolotti@uniba.it (O.N.); 2 Department of Chemistry, Biology and Biotechnology, University of Perugia, Via Elce di Sotto, 8, 60123 Perugia, Italy; gabriele.cruciani@unipg.it; 3 Molecular Discovery Ltd., Kinetic Business Centre, Theobald Street, Elstree, Borehamwood, Hertfordshire WD6 4PJ, UK; gabo@moldiscovery.com; 4 Molecular Horizon srl, Via Montelino 30, 06084 Bettona, Italy

**Keywords:** ligandable pockets, 3D oncoPPI networks, PPIs modulators, PROTACs, pocketome analysis, target prioritization in cancer

## Abstract

**Background/Objectives:** Studying protein–protein interaction (PPI) networks is crucial in understanding cancer phenotypes and molecular mechanisms. Here, we focus on PPIs involved in 12 different types of cancer (oncoPPIs), highlighting those protein pockets serving as outposts to modulate protein functioning. **Methods:** To explore these cavities linked to the cancer phenotype changes, we built a comprehensive pocketome of 314 crystallographically solved oncoPPIs. Based on this experimental data, we identified and investigated all ligandable protein pockets by employing 3D geometric and energetic descriptors. These pockets were classified as suitable for designing new oncoPPI modulators or PROTACs. The ligand-bound crystallographic pockets were analyzed to compare their properties across cancer types. Finally, 3D oncoPPI networks were built for each cancer type to identify highly connected proteins acting as hubs. **Results:** Combining interaction networks with structural pocket data helps identify cancer-relevant proteins and key interacting residues. Using this approach, we present clinical examples (e.g., S100A1, NRP1, CTNNB1, VCP) to show the therapeutic value of targeting ligandable 3D oncoPPIs. We also provide a publicly available reference dataset supporting future research. **Conclusions**: Notably, this study offers a flexible framework for evaluating and prioritizing novel disease targets.

## 1. Introduction

Nowadays, cancer remains one of the major issues for medical research due to its vast impact on society. Almost half of global cancer cases occur in Asia (49.2%), mainly in Eastern Asia including China, followed by Europe (22.4%) and the Americas (21.1%). Cancer deaths are also highest in Asia (56.1%), with Europe (20.4%) and the Americas (14.9%) trailing behind [1]. Cancer leads to an annual cost of EUR PPP 449 billion for health systems, representing a 6% rise in healthcare spending compared to a scenario without cancer [2].

One of the primary challenges in cancer biology revolves around comprehending the transformation of a normal cell into a cancer cell. Nowadays, it is evident that protein–protein interactions (PPIs) play a significant role in all the stages of cancer onset and progression. They are involved in the signaling pathways governing cell proliferation, growth, apoptosis, differentiation, and metabolism. Consequently, developing modulators that interfere with PPIs and targeting specific biochemical pathways of oncoproteins could be a key strategy in molecular cancer therapy [3,4,5].

Enhanced comprehension of the protein interaction networks and specific protein hubs, within each cancer type holds significant promise in advancing personalized medicine approaches. For this reason, there is an urgent need for a cancer-specific view of PPIs. Several studies have explored cancer-related PPIs using structural and network-based approaches. Kamburov et al. [6] mapped missense mutations onto 3D protein structures to identify novel cancer proteins and functional mutations in PPIs. Li et al. [7] later developed oncoPPIs, experimentally characterizing over 260 cancer-associated PPIs in lung cancer cells. Large-scale pan-cancer analyses further classified differential PPIs, distinguishing activated and repressed interactions [8], while platforms like PINA [9] integrated multi-omics data for cancer type-specific PPI investigations. Techniques such as single-molecule tools and fluorescence resonance energy transfer measurements are widely exploited to measure the mechanical strength of biomolecular complexes, including PPI systems, and to provide a quantitative characterization of their interaction properties [10,11].

In silico methodologies have accelerated the study of oncoprotein interfaces for drug discovery. Integrating 3D structures into PPI networks has revealed distinct features of cancer proteins, such as smaller and more charged binding sites [12]. Proteins overexpressed in 10 tumor types, but not necessarily involved in PPIs, were mapped in the search of putative interaction sites (i.e., catalytic sites, PPI sites, and unclassified sites) [13].

Targeting PPIs has become more attractive, since the general assumption that PPI interfaces are undruggable (large, flat, and featureless) has been overcome as demonstrated by numerous PPI-specific inhibitors that have entered clinical trials for cancer and some already on the market [14].

Furthermore, the PROTAC (proteolysis targeting chimera) route is a fascinating alternative for overcoming the limits of resistance and high-dose requirements, and thus for targeting what is sometimes defined as “undruggable”, or more accurately, “difficult to drug” [15]. A PROTAC is a molecule designed to eliminate specific unwanted proteins. PROTACs as anticancer therapies have been developed in recent years [16,17], capturing several oncoproteins such as nuclear receptors [18], kinases [19], transcriptional regulators [20], and others [21].

In this respect, it is thus important (a) to look at protein networks from a cancer-specific perspective; (b) to employ in silico methodologies for investigating and exploring oncoproteins and oncoPPIs in a three-dimensional manner; and (c) to explore oncoPPIs modulators and PROTACs, as well as, more generally, allosteric inhibitors, for designing new effective therapies. In this study, we aimed to design a strategy that addresses these needs. The main aim was to put a magnifying glass on key protein pockets that can regulate and modulate oncoproteins and oncoPPIs. This methodology, previously successfully applied to other case studies of 3D protein pocket mapping [22,23,24,25], employs GRID-based approaches [26] to pinpoint areas within the protein structure that could either be significantly buried or relatively exposed while still exhibiting features suitable for interacting with drug-like small molecules.

To the best of our knowledge, we present for the first time an unprecedented, comprehensive compendium of pockets derived from 3D crystallographic structures of oncoPPIs activated across 12 distinct cancer types, based on targets identified in Gulfidan et al. [8]. For each oncoPPI uniquely activated in tumors versus healthy tissue, we performed two critical analyses: (a) we identified interface pockets with immense potential for the design of oncoPPI modulators, and (b) we uncovered additional surface pockets that can be exploited as anchoring sites for PROTAC-mediated degradation of one of the interacting partners.

Our in silico protocol [27] was validated with a success rate of 75% and 100% for known cases of oncoPPIs inhibitors and PROTAC binding sites, respectively. All the potential pockets, both on the interface of 3D oncoPPIs and on other favorable protein regions for PROTAC design, were thus mapped. Based on molecular interaction fields (MIFs) [26], the geometric and energetic physicochemical properties were inspected in order to have a clear picture of the content of these pockets. To assess their ligandability content, we examined and characterized crystallographic ligands already known to bind them. Furthermore, the occurrence of analyzed ligands was different across various cancer types.

Notably, we also constructed 3D oncoPPI networks to identify hub proteins, which are therapeutically relevant for each cancer type due to their high degree of connection to other proteins. This study focuses on the structural features of proteins and pockets responsible for oncoPPIs, paving the way to prioritize targets with high druggability content. This approach aims to streamline the rational design of new drugs and to suggest new therapeutic strategies. For instance, we identified promising pockets for drug design in various oncoproteins and oncoPPIs, including S100A1, HIF1α, MTOR, NRP1, CTNNB1, and VCP. Notably, for the first time, a 3D oncoPPI comprehensive reference dataset of protein and pocket structures is provided. The data are freely accessible to the scientific community at https://github.com/moldiscovery/OncoPPI-pocketome, accessed on 14 May 2025.

The novelty of our work lies in the introduction of a 3D standpoint of oncoPPIs. Unlike previous studies, we focus on the binding pockets involved in cancer-associated PPIs, as these regions are of particular interest for drug design. For the first time, these PPIs are characterized from a three-dimensional and drug-oriented perspective, describing the structural regions and interactions involved in the binding of putative ligands.

In addition, we propose a flexible framework for systematically assessing and ranking emerging disease targets, enabling the identification of critical proteins in diseases and offering a structured approach to their evaluation.

## 2. Results and Discussion

### 2.1. Detection of Known oncoPPI Inhibitor and PROTAC Binding Pockets

**Figure 1:**
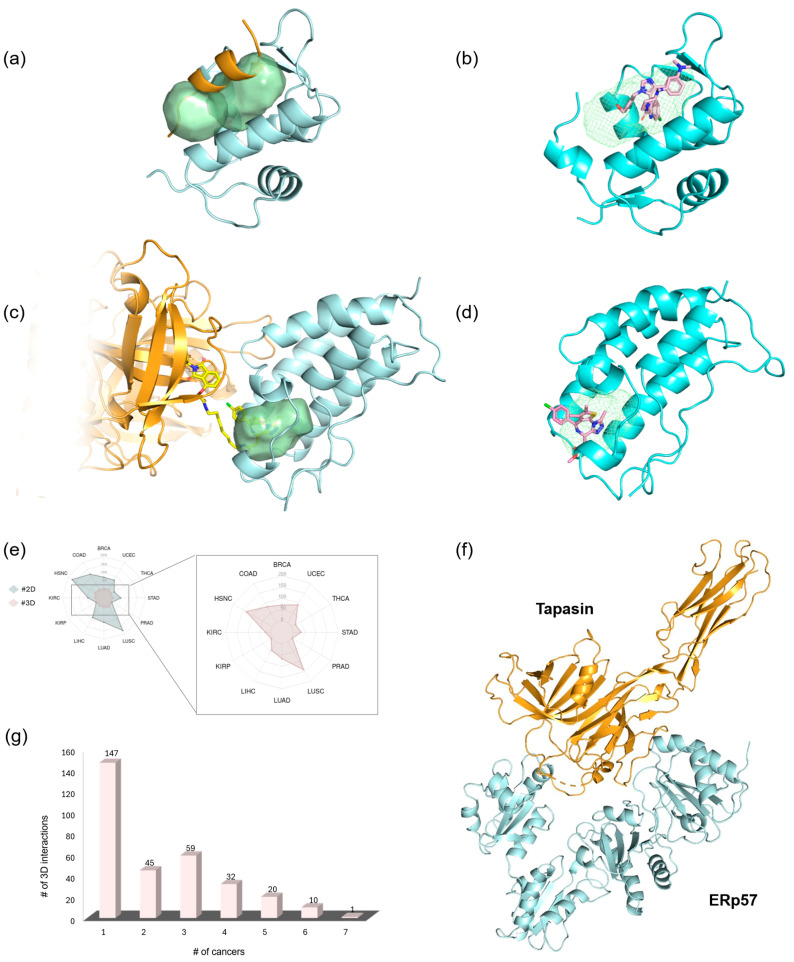
Successful examples of predicted pockets in validation dataset. (**a**) Pocket at the interface of HDM2/P53 (PDB ID: 1ycr). (**b**) Pocket detected on HDM2 co-crystallized with inhibitor HTZ (PDB ID: 6q9l). (**c**) Pocket at the interface of CRBN/BRD4 with RN6 PROTAC (PDB ID: 6boy). (**d**) Pocket detected on BRD4 co-crystallized with inhibitor 0S6 (PDB ID: 4f3i). (**e**) Spider plots reporting the number of activated PPIs in Gulfidan et al. [8] work (2D-act, in grey) and the number of mapped activated interactions in PDB (3D-act, in pink). (**f**) The crystallographic complex of the most recurrent 3D oncoPPI, namely Erp57 and Tapasin (PDB ID: 3f8u). (**g**) Histogram of the number of activated 3D oncoPPIs per cancer. Pockets detected are displayed as surface and as mesh in the protein-bound form and in the ligand-bound form, respectively. Protein interactors are colored in cyan and orange cartoon, whereas ligands and PROTAC are colored in yellow and pink, respectively.

First, we explored known cases of (a) inhibitors disrupting oncoPPIs and (b) PROTACs in cancer therapies, testing BioGPS’s [27] ability to guess the protein cavities hosting potential binding sites. Data were collected from literature [16,28,29,30] and the Protein Data Bank (PDB), resulting in two validation sets: (a) 16 oncoPPIs and (b) 7 PROTAC complexes (Table S1). BioGPS detected protein pockets at oncoPPI interfaces in 81% of cases (13/16), and 75% when one protein was bound to a disruptor (12/16). For the PROTAC set, BioGPS successfully identified pockets in all seven cases, both for PROTAC-bound and inhibitor-bound proteins. Examples of protein-bound and ligand-bound for both these pools are reported in Figure 1. Furthermore, we evaluated the accuracy in detecting pocket residues by comparing the known protein residues interacting with the ligand/partner and the residue defining the pocket by calculating the Matthew correlation coefficient (MCC) [31] (Table S1). MCC values ranged from 0.58 to 0.90, thus proving the outstanding accuracy in detecting pockets for targeting oncoPPIs and for PROTAC-related protein systems.

### 2.2. 3D Dataset of oncoPPIs

In order to address the design of potential inhibitors or modulators of these oncoPPIs, we focused on the so-called “activated” interactions occurring in the cancerous state, leaving aside for the moment those “repressed” [8,28].

The paper aims to create a 3D data collection for drug design. Starting with PPIs from cancerous tissues by Gulfidan et al. [8], we searched the PDB for these interactions to study them from a 3D perspective. The protein interactions derived from this analysis will henceforth be called 3D oncoPPIs. The 12 human cancer types analyzed in the study are as follows: breast invasive carcinoma (BRCA), colon adenocarcinoma (COAD), head and neck squamous cell carcinoma (HNSC), kidney renal clear cell carcinoma (KIRC), kidney renal papillary cell carcinoma (KIRP), liver hepatocellular carcinoma (LIHC), lung adenocarcinoma (LUAD), lung squamous cell carcinoma (LUSC), prostate adenocarcinoma (PRAD), stomach adenocarcinoma (STAD), thyroid carcinoma (THCA), and uterine corpus endometrial carcinoma (UCEC). A detailed report of their mapped interactions (both activated and repressed) is reported in Table S2 of the Supporting Information.

Gulfidan et al. [8] identified 4671 activated interactions in cancer, involving 1967 proteins. In a 3D context, 314 activated oncoPPIs were found, involving 332 crystallographic proteins. Despite their importance, only a limited number of crystallographic structures are available (Figure 1e, left-hand side; see Table S2 in the Supporting Information). The study focuses on 7% of the total data (314 out of 4671), but the availability trend is consistent across cancer types, confirming the 3D data pool represents a robust subset of the overall data. As is well known, mutations in certain proteins can lead to the promotion or suppression of PPIs, thereby influencing cancer progression [32]. Notably, the crystallographic structures used so far have not been selected based on the presence or absence of mutations. However, a potential future direction could involve analyzing how the pocket shape and interactions involved in oncogenic PPIs change in response to variations in key residues.

The first question we tried to answer is whether some 3D oncoPPIs were activated in more than one cancer type, making them more recurrent indicators of a tumor state. In about half of the cases (147/314), the oncoPPIs were activated only in one type of cancer (Figure 1g). In the remaining cases (167/314), the oncoPPI appeared to be activated in several types of cancer. In particular, we report in Figure 1f the most frequent oncoPPI, an activated interaction between Tapasin and ERp57, highlighting the role of ERp57 in cancer growth and progression [33]. This interaction was found to be a crucial interaction in BRCA, HNSC, KIRC, LIHC, LUAD, THCA, and UCEC.

### 2.3. 3D Protein Pocket Mapping: Development of the oncoPPI Pocketome

**Figure 2:**
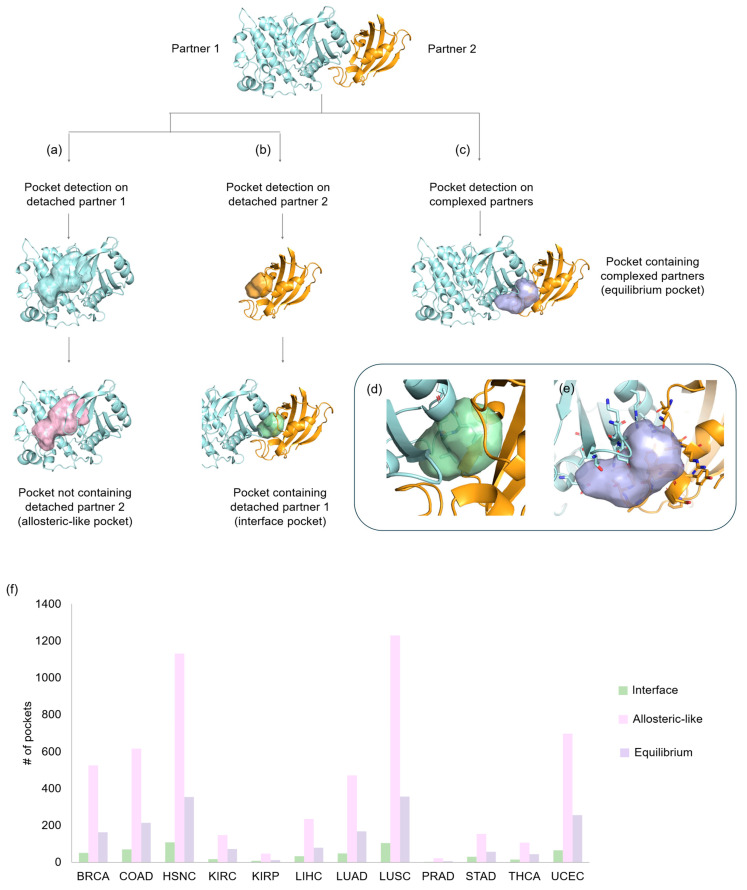
(**a**) Pocket detection on detached partner 1 (cyan), resulting in an *allosteric-like* pocket (pink surface) that does not include the other partner. (**b**) Pocket detection on detached partner 2 (orange), resulting in an *interface* pocket (green surface) that contains the other partner. (**c**) Pocket detection on the complexed partners, resulting in an *equilibrium* pocket (violet surface) defined by both partners. In the inset, a zoomed-in detail highlights the following: (**d**) on the left, the *interface* pocket (green surface) defined by residues of detached partner 2 and containing an alpha helix from detached partner 1 (cyan sticks), and (**e**) on the right, the *equilibrium* pocket (violet surface) defined by residues of both partners (orange and cyan sticks). (**f**) Histogram showing the number of pockets per cancer for each category.

The proposed method detects potential binding sites by analyzing both the spatial configuration and the physicochemical properties that promote such binding [27]. Starting from activated 3D oncoPPIs, we first extracted the corresponding crystallographic complexes from the PDB (see Materials and Methods). We then searched for pockets in two different ways on these complexes (Figure 2).

1. Detached partners: the search for pockets was carried out on each individual partner separately by splitting the crystallographic complex, so that any pocket at the interaction interface could be revealed for both partners (Figure 2a,b).
2. Complexed partners: in this case, we analyzed the interaction zone of the whole complex in order to identify pockets involved in the bound/unbound equilibrium (Figure 2c).

In this, we obtained three categories of pockets:

- *Interface* pockets: these pockets were identified on individual detached partners and are located in regions involved in 3D oncoPPIs. A pocket is classified as an *interface* pocket if the protein residues of the interacting partner are enclosed within it (Figure 2d).
- *Allosteric-like* pockets: these pockets are found on individual detached partners and do not correspond to regions directly involved in 3D oncoPPIs.
- *Equilibrium* pockets: these pockets were computed on the complexed partners and are located in regions defining the interaction interface between the two protein partners. To be classified as an *equilibrium* pocket, it must consist of residues belonging to both interacting partners (Figure 2e).

The number of the detected pockets, classified into the three categories (*interface*, *allosteric-like*, *equilibrium*), for each cancer type is reported in Figure 2f (details are available in Table S3).

#### 2.3.1. Interface and Allosteric-like Pockets on Detached Partner

Based on 314 crystallographic complexes of activated oncoPPIs, we detected at least one pocket in 310 cases; at least one pocket at the interface of the two partners (*interface* pockets) in 142 cases; and at least one pocket located on other regions of the protein (*allosteric-like* pockets) in 296 cases. In total, we collected 2800 pockets, comprising 248 *interface* pockets and 2552 *allosteric-like* pockets.

##### Interface Pockets

Among 142 3D oncoPPIs with *interface* pockets, 22 involve homo-interactions, while 120 are hetero-interactions. Of the latter, 87 have a pocket on one partner, while 33 have pockets on both partners (Figure S1).

**Figure 3:**
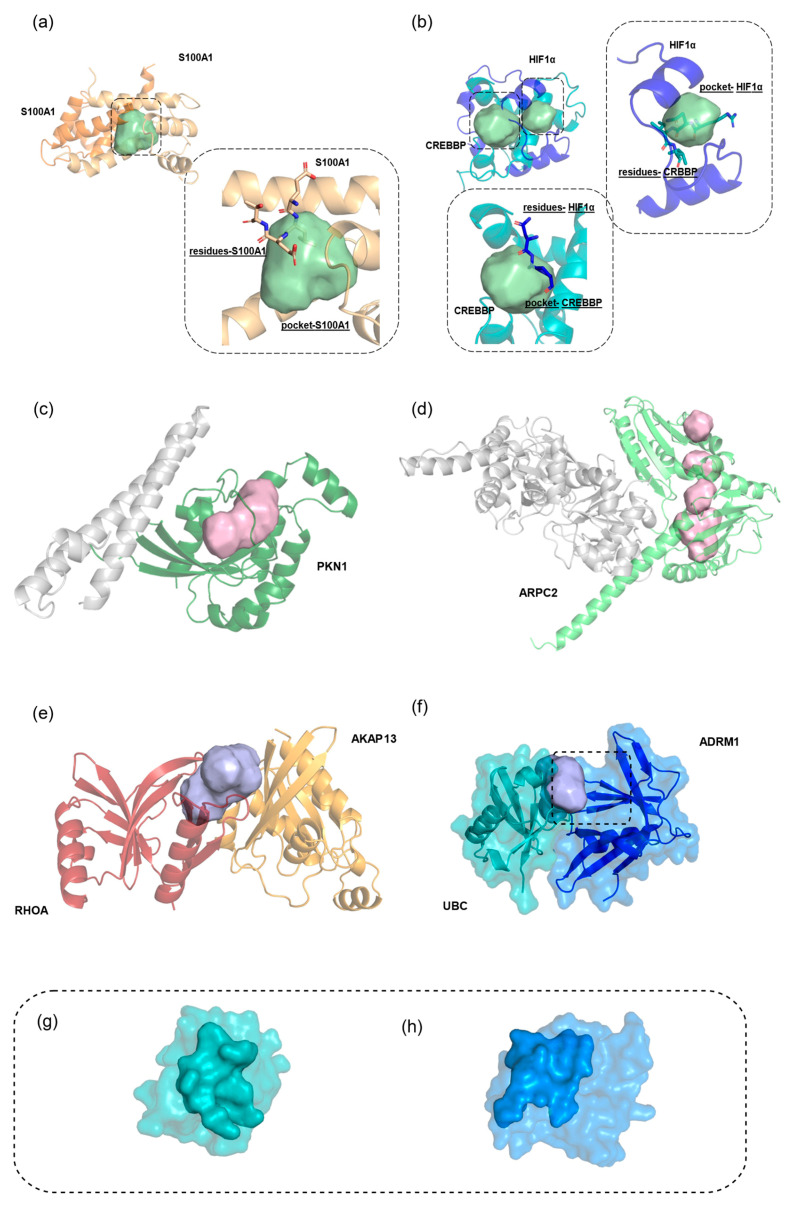
*Interface* (**a**,**b**), *allosteric-like* (**c**,**d**), and *equilibrium* (**e**–**h**) pockets. (**a**) The S100A1 homodimer is shown with two monomers in different shades of orange. The enlarged region highlights a pocket on one monomer that contains residues from the other (light orange sticks) (PDB ID: 5k89). (**b**) In the HIF1α–CREBBP heterodimer, HIF1α is depicted in blue and CREBBP in cyan. Two enlarged views are provided: (**left**) one shows a pocket on CREBBP containing HIF1α residues (blue sticks) and (**right**) the other shows a pocket on HIF1α containing CREBBP residues (cyan sticks) (PDB ID: 1l8c). (**c**) PKN1 is illustrated with its *allosteric-like* pocket, shown as a dark green cartoon with a pink surface; the interacting partner is represented in light grey (PDB ID: 1cxz). (**d**) ARPC2 displays its *allosteric-like* pockets as a light green cartoon with a pink surface, with the interacting partner in light grey (PDB ID: 6uhc). (**e**) The RHOA–AKAP13 heterodimer exhibits an *equilibrium* pocket (violet surface) that includes residues from both proteins. RHOA and AKAP13 are shown in red and yellow, respectively (PDB ID: 6bca). (**f**) The UBC–ADRM1 heterodimer features an *equilibrium* pocket (violet surface) encompassing residues from both partners highlighted in black dotted box (PDB ID: 5v1y). (**g**,**h**) A zoomed-in view of the UBC–ADRM1 heterodimer highlights the surface portion interacting with the *equilibrium* pocket: UBC residues interacting with ADRM1 are depicted as a dark cyan surface, while ADRM1 residues interacting with UBC are shown as a dark blue surface.

One of the first cases of homodimeric interactions that caught our attention involves the S100A1 protein, a calcium-binding protein belonging to the S100 family shown to be overexpressed in THCA [8,34]. It has been demonstrated that the knockdown of S100A1 dramatically inhibits cell proliferation and migration, making it a key protein for the diagnosis and prognosis of thyroid carcinoma. This study offers the opportunity to identify the molecular initiating event for potential inhibition of the dimerization of this protein. Indeed, the identified pocket is located at the dimer interface and represents a potential binding site for disruptors of this dimerization (Figure 3a).

An interesting case of a heterodimer, with pockets identified on both partners (Figure 3b), is the interaction between Hypoxia-inducible factor 1-alpha (HIF1α) and CREB-binding protein (CREBBP), overexpressed in HNSC. Transcriptional regulation by HIF-1α relies entirely on the interaction between its C-terminal activation domain (CAD) and the TAZ1 domain of CREBBP. Attempts to disrupt this interaction have been previously reported, with the antibiotic novobiocin used as a strategy to inhibit hypoxic responses in tumors by blocking the PPI between HIF1α and CREBBP [35]. In our study, we identified two cavities, one on each partner, further expanding the possibilities for preventing this interaction.

Another example of a heterodimer, but with a pocket on one of the two partners is represented by the E3 ubiquitin–protein ligase Itchy homolog (ITCH) and the thioredoxin-interacting protein (TXNIP). The pocket, detected on ITCH, contains residues from partner TXNIP, meaning that a modulator mimicking the interactions of these residues could potentially act as an inhibitor [36] (Figure S1).

##### Allosteric-like Pockets

The 296 complexes in which at least one *allosteric-like* pocket was identified consist of a total of 315 proteins. Some of these proteins contain only a single pocket (not located at the interface), while others feature multiple pockets (Figure 3c,d).

The PKN1 protein shows an overexpression of its interaction with certain partners (vimentin and transforming protein RhoA) in various cancer types (i.e., KIRC, LUSC, UCEC, LUAD). Additionally, it has been identified as a key protein in promoting liver cancer proliferation [37]. This protein features a single allosteric-like site (Figure 3c), which is potentially useful for PROTAC design, as it is also sufficiently exposed to the solvent. Degradation of this protein could lead to the elimination or reduction of its overexpressed interaction with its partners. Similarly, on the actin-related protein 2/3 complex subunit 2 (ARPC2), which interacts with actin-related protein 3 in LUSC, four potential sites have been identified that could be useful for PROTAC design (Figure 3d). On the other hand, some cases may prove more challenging due to the abundance of clefts, complicating the target triage process (DNA-dependent protein kinase catalytic subunit (PRKDC) in Figure S1).

#### 2.3.2. Equilibrium Pockets on Complexed Partners

Focusing on the pockets found in the interaction region between the two partners, we identified a total of 812 pockets across 262 out of 314 oncoPPIs crystallographic complexes. These pockets form only when the two partners interact, thus holding a different biological meaning from the interface pockets previously discussed. Given their transient nature, these are referred to as *equilibrium* pockets, and they exist based on the equilibrium of the bound and unbound forms. Again, we have categorized the pockets into cases where they are formed by homodimers (39 out of 262) or heterodimers (223 out of 262, Figure S1). The percentage of residues from both partners that define an *equilibrium* pocket is reported in Table S4 of the Supporting Information. For example, some pockets are defined equally by 50% of residues from each partner, while others are characterized by a higher percentage of residues from a given single partner.

An interesting example of *equilibrium* pockets is found in Rho GTPases, key regulators of cellular processes in UCEC cancer [8]. While a known small-molecule inhibitor targets the AKAP13-RhoA interaction at the DH domain [38], our study highlights a ligandable pocket at the PH domain. By targeting this specific pocket, a ligand could stabilize either the bound or unbound form, offering a potential mechanism to influence the protein functional dynamics. The most interesting aspect of the *equilibrium* pockets dataset is that the interactions, where a pocket was detected, have nearly doubled compared to the detached *interface* pockets cases (262 vs. 142). The proteasomal ubiquitin receptor ADRM1 and ubiquitin (UBC) (Figure 3f), involved in COAD, HNSC, and LUSC, represent an example of interactions based on an *equilibrium* pocket whose *interface* is unidentifiable considering the detached partners only. In this case, the two regions contributing to the interface are relatively flat when considered individually (Figure 3g,h) and do not contain clefts suitable for the formation of binding pockets. However, upon complex formation, a cleft is formed adjacent to the interaction zone, thus creating a potential binding site for modulators of this 3D oncoPPI (Figure 3f). This newly formed pocket gives the chance to target such an interaction with small drug-like molecules designed to stabilize or disrupt the formed complex.

### 2.4. Ligandable Pockets for 3D oncoPPI Modulators and PROTAC Design

The pockets identified in our study represent a vast collection of potential targets. A crucial factor to consider is whether a ligand has been specifically designed to engage a given pocket within a protein. To address this, we conducted a comprehensive analysis of all known ligands available in the PDB to determine whether any were localized within the pockets of the proteins in our benchmark dataset. This expanded approach enabled us to classify cancers, proteins, and pockets based on their prior ligandability, providing a critical foundation for drug design efforts.

As part of our analysis, we have identified a total of 219 ligand-bound pockets in detached partners, which include 19 *interface* pockets out of 248 (7.7%) and 138 *allosteric-like* pockets out of 2552 (5.4%). Additionally, we found that 62 *equilibrium* out of 812 (7.6%) are ligand-bound in complexed partners. The total number of detected crystallographic ligands is 667. The data collected emphasizes the potential for further exploration of these ligand-bound pockets in drug design and therapeutic applications.

**Figure 4:**
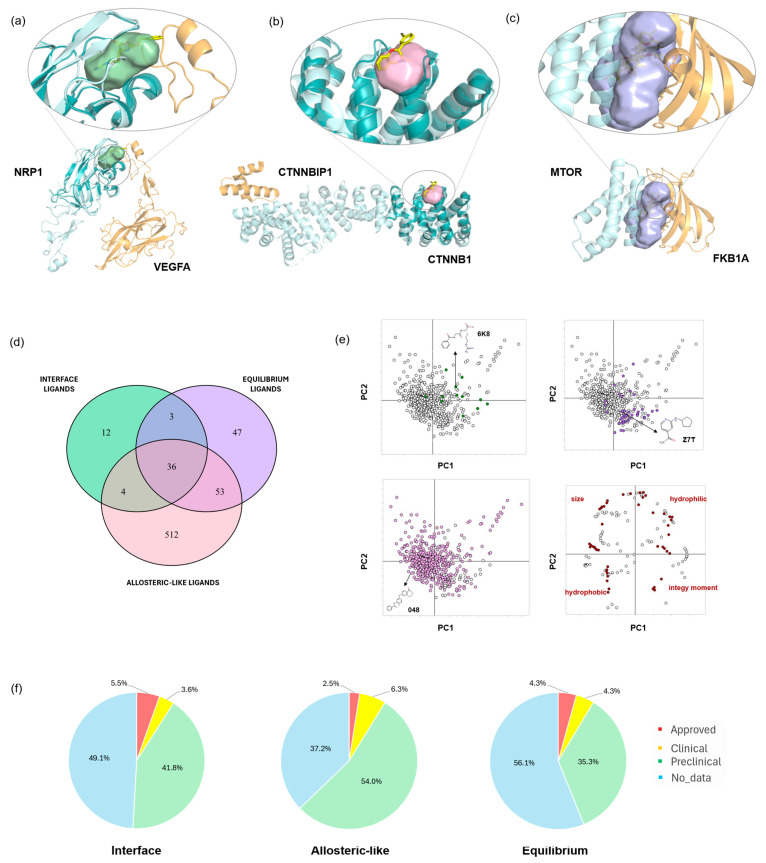
Examples of ligand-bound pockets showing specific binding regions where ligands interact with the protein. (**a**) *Interface* pocket in green detected on the detached partner NRP1 (cyan cartoon) in complex with VEGFA (orange cartoon) (PDB ID: 4deq). The detached partner NRP1 (dark cyan cartoon) is complexed with compound R40 (yellow stick) (PDB ID: 5iyy). (**b**) *Allostericlike* pocket in pink detected on the detached partner CTNNB1 (cyan cartoon) in complex with CTNNBIP1 (orange cartoon) (PDB ID: 1t08). The detached partner CTNNB1 (dark cyan cartoon) is complexed with compound R9Q (yellow stick) (PDB ID: 7afw). (**c**) *Equilibrium* pocket in violet found in the region between the FKBP1A and FKBP12–rapamycin-associated protein (MTOR) (PDB ID: 3fap) with rapamycin analog ARD (yellow stick) interacting simultaneously with the complexed partners. (**d**) Venn–Eulero diagram of crystallographic ligands found within *interface*, *allosteric-like*, and *equilibrium* pockets. (**e**) PCA scores and loadings plots showing the crystallographic ligands found within *interface*, *allosteric-like*, and *equilibrium* pockets. Ligands from *interface* pockets, such as 6K8, are marked in green; *equilibrium* pocket ligands, such as Z7T, in purple; and *allosteric-like* pocket ligands, such as 048, in pink. The lower right corner displays the PCA loadings plot, highlighting descriptors related to shape/size as well as hydrophobicity and hydrophilicity. Explained variance: PC1: 33.66%–PC2: 24.31%. (**f**) Pie chart reporting the classification of the clinical status of ligands found in the different categories of pockets.

A ligand-bound *interface* pocket was identified on neuropilin-1 (NRP1), a VEGFA coreceptor involved in angiogenesis in KIRC cancer [8]. The pocket, located at the VEGFA-NRP1 interface (Figure 4a), is an ideal target for disruptors. Existing NRP1 antagonists that bind this site could be optimized to inhibit VEGFA interaction or serve as moieties for PROTAC design, enabling targeted NRP1 degradation as a novel anticancer strategy.

A ligand-bound *allosteric-like* pocket was found in catenin beta-1 (CTNNB1), which is overexpressed in COAD [8]. The identified pocket, not involved in PPIs, has been shown to bind known ligands [39], making it a promising target for PROTACs. Given the challenges in developing potent CTNNB1 inhibitors, this approach offers a potential breakthrough for selective protein degradation (Figure 4b).

A ligand-bound *equilibrium* pocket was identified at the FK506-binding protein (FKBP1A) and the FKBP12–rapamycin-associated protein (MTOR) interface, an interaction activated in STAD [8]. Rapamycin, a cell-cycle arrest agent, binds both proteins simultaneously, with MTOR inhibition driving its therapeutic effects. This dual binding mechanism underscores the importance of equilibrium pockets in drug design, as it illustrates how the simultaneous engagement of multiple sites can lead to significant biological outcomes (Figure 4c).

After collecting all the crystallographic ligands within the identified pockets (by aligning all available crystallographic structures, see Methods), we analyzed which pockets currently have the highest ligand occupancy, as these might represent the most ligandable sites (Table 1).

The ligandability potential of the identified pockets was further assessed through an in-depth analysis of the physicochemical properties of their crystallographic ligands [40]. For this analysis, ligands were classified by considering only those found exclusively in a single pocket category (Figure 4d), discarding those shared across multiple categories (e.g., ligands present in both *allosteric-like* and *interface* pockets). Comprehensive information on all ligands identified within the detected pockets is available in Table S5 of the Supporting Information.

Crystallographic ligands in these pockets share many properties, displaying a range of molecular weights (32.04–1529.83 Da) and polarity (3.016 Å^3^–149.684 Å^3^). Notably, equilibrium pocket ligands (Figure 4e, top right) tend to cluster in the PCA plot within a region dominated by smaller and more polar molecules. These often exhibit a high “integy moment” (vector pointing from the center of mass to the center of the hydrophilic regions), which is a characteristic of ligands with hydrophilic regions concentrated on specific areas of the molecular surface. These properties align well with those expected for modulators of PPIs, molecular glues, and other molecules designed to interact across both partner surfaces within PPIs. Ligands occupying the *interface* and the *allosteric-like* pockets, on the other hand, share a common spatial region (Figure 4e, top and bottom left-hand side), further demonstrating that PPI inhibitors possess characteristics closely aligned with those of drug-like small molecules targeting more deeply buried pockets. To assess the therapeutic relevance of ligands, we analyzed DrugBank [41] and ChEMBL [42] data, including ATC codes (Table S6 of the Supporting Information). Among 667 crystallographic ligands, 17 (2.5%) were found to be in the *approved* category, mainly as antineoplastics (e.g., ixazomib, vemurafenib, sorafenib). Additionally, 40 ligands are classified as *clinical*, with 18 classified as potential antitumor agents. The remaining ligands were categorized as *preclinical* or lacked available data (Figure 4f).

**Figure 5:**
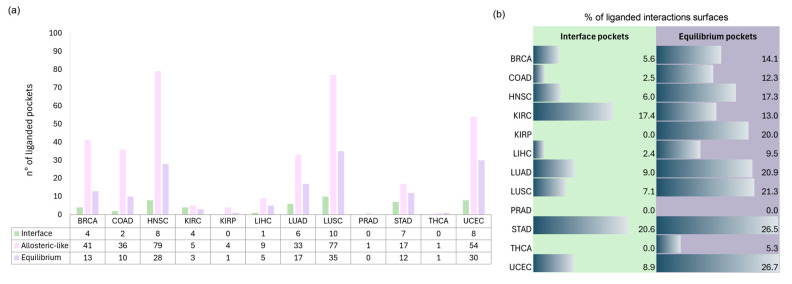
(**a**) Distribution of ligand-bound pockets across various cancer types. The *y*-axis shows the number of ligand-bound pockets for each of the three pocket categories: *interface* (green bars), *allosteric-like* (pink bars), and *equilibrium* (violet bars). (**b**) Data bar representing the percentage of 3D oncoPPIs surfaces where at least one ligand-bound pocket was found, both with *interface* pocket and with *equilibrium* pocket.

We analyzed ligandability across different cancer types, focusing on those with the highest number of ligand-bound pockets. As shown in Figure 5a, efforts have primarily targeted HNSC and LUSC, reflecting their high number of interactions and proteins. Across all cancer types, *allosteric-like* pockets dominate, as they span various regions of the protein rather than being confined to interaction interfaces. Interestingly, *equilibrium* pockets contain more ligands than interface pockets (Figure 5a, violet vs. green bars), suggesting that many ligands bind at sites influenced by both interacting partners rather than a single protein. To assess which cancers have ligand-bound 3D oncoPPIs, we calculated the percentage of interactions with at least one ligand at the interface (both *interface* and *equilibrium* pockets, Figure 5b). Notably, STAD, KIRC, and UCEC show the highest percentages, highlighting potential opportunities for targeting specific PPIs using existing ligands, which could facilitate therapeutic interventions.

This analysis underscores a vast potential for drug design campaigns. Many targets already have crystallographic ligands, and additional non-crystallographic ligands likely exist in the literature.

Due to the immediacy of the mechanism of action of the *interface* and *equilibrium* pockets, with the latter being slightly more ligandable than the interface (Figure 5a), we consider these to be promising starting points for the design of antitumor drugs. Starting from known ligands, optimization can be carried out using structure-based approaches, as the pocket environment is well characterized through MIFs. Nevertheless, it is worth noting the extensive availability of known ligands for *allosteric-like* pockets, which makes them attractive starting points for PROTAC design. This strategy may also help overcome some of the limitations of classical inhibitors, such as issues related to dosage and resistance.

### 2.5. Geometric and Energetic Anatomy of the 3D oncoPPI Pocketome

To investigate the physicochemical space of pockets identified in activated 3D oncoPPIs, both ligand-bound and unbound, we calculated a series of descriptors to analyze their anatomical features. The primary question we sought to address was whether ligand-bound pockets share characteristics with those that are not bound to ligands. Such a finding would suggest that there is significant untapped potential for drug design within these 3D oncoPPIs, underscoring the necessity of further exploring and navigating this vast space.

**Figure 6:**
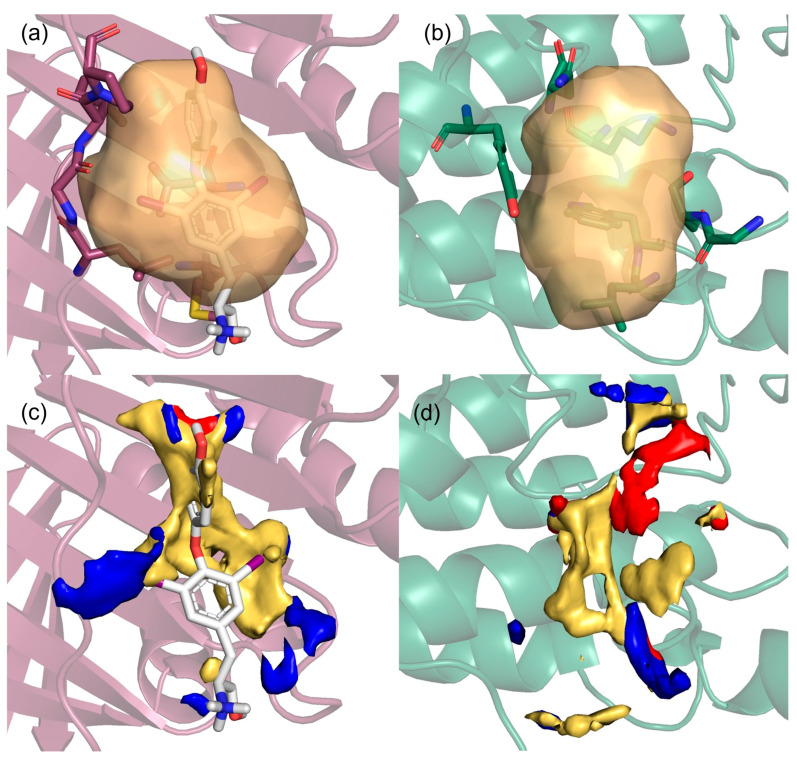
(**a**–**c**) *Interface* pocket of the PCNA protein dimer interaction (PDB ID: 1u76), with a T2B ligand extracted from a holo structure (PDB ID: 3wgw) and the corresponding MIFs. (**b**,**d**) *Allosteric-like* pocket on BRAF (PDB ID: 6u2h) and the corresponding MIFs. Protein and pocket residues defining the pocket are displayed as cartoon and stick representations, respectively. The ligand bound is displayed as grey stick representations. Hydrophobic, hydrogen bonding donor, and hydrogen bonding acceptor MIFs are displayed as yellow, blue, and red surfaces, respectively. The probes used are as follows: CRY for hydrophobic (energy threshold = −1.0 Kcal/mol), N1 for hydrogen bonding donor (energy threshold = −4.5 Kcal/mol), and O for hydrogen bonding acceptor (energy threshold = −4.5 Kcal/mol).

The distribution of descriptors reveals that, for all three pocket categories, a significant proportion of the discovered pockets share morphological features (such as shape rugosity and globularity) with ligand-bound pockets (Figure S2). Moreover, an analysis of the balance between hydrophobic and hydrophilic interactions unveils instances across all pocket categories that exhibit similar anatomy to the ligand-bound pockets. An illustrative case of this behavior is depicted in Figure 6, and relative GRID-based molecular descriptors are summarized in Table 2.

The pocket at the interface of the proliferating cell nuclear antigen (PCNA) protein dimer interaction (Figure 6a) is a well-known region for binding ligands, such as the T2 amino alcohol (T2AA) [43]. This pocket exhibits morphological and energetic characteristics similar to an *allosteric-like* pocket (Figure 6b) on serine/threonine-protein kinase B-raf (BRAF). The latter pocket has not yet been reported as ligand-bound in the PDB, and it does not correspond to a well-known binding site in kinases [44]. Both pockets have a volume of approximately 800 Å^3^. The values for rugosity, globularity, hydrophilic volume, hydrophobic volume, solvent exposure, and buriedness are remarkably similar, as reported in Table 2. Moreover, when analyzing the maps generated with MIFs [27], it is evident that the spatial arrangement of the two pockets shares common features, such as a central hydrophobic core and polar anchoring points (Figure 6c,d). This type of analysis can be useful in identifying pockets that have not yet been studied as potential ligand-binding sites but share characteristics with known ligand-binding pockets.

### 2.6. Hub Proteins and Hub Pockets in 3D oncoPPI Networks

Hub proteins, highly connected within PPI networks, are crucial regulators of cellular processes and are often overexpressed in cancer, driving tumor growth and survival [45]. Their central role makes them key therapeutic targets, as inhibiting them could disrupt multiple oncogenic pathways simultaneously. In the context of cancer, analyzing hub proteins is particularly important for several reasons, such as network robustness and vulnerabilities, biomarker discovery, potential therapeutic targets, and resistance mechanisms.

In the following section, we identify hub proteins, defined as proteins interacting with two or more partners, within the analyzed 3D oncoPPIs from a structural perspective, highlighting their prevalence across various cancer types. We then focus on a specific hub pocket, a key region directly contributing to network centrality.

**Figure 7:**
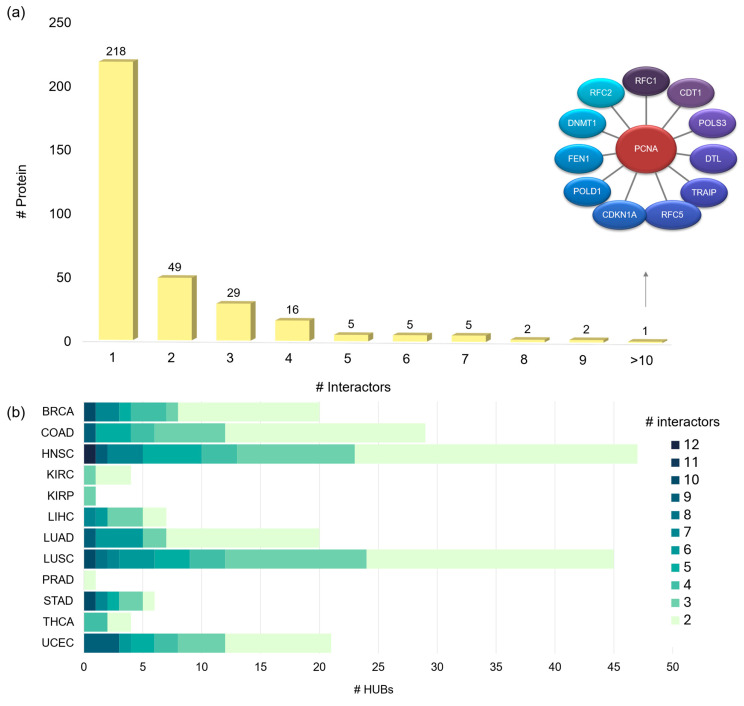
(**a**) Distribution of the number of interactors per protein. This histogram illustrates the frequency of proteins based on the number of crystallographic interactors they engage with, highlighting the distinction between single-interactor proteins and multi-interacting hub proteins across the dataset. Network of the most populated 3D oncoPPI crystallographic hub (PCNA). (**b**) Histogram of hub proteins per cancer type. Each bar represents the number of hub proteins identified in each cancer type. The color gradient within the bars reflects the degree of connectivity, ranging from hubs with fewer interactors (light green) to those with extensive interaction networks (dark green).

Among 314 3D oncoPPIs, we identified 332 unique proteins. To assess their connectivity, we calculated the total number of crystallographic interactions for each protein, independent of cancer type (Figure 7a). 

We define a hub protein as one that engages in two or more interactions (≥2) within the 3D oncoPPI network. A total of 114 proteins are classified as hubs due to multiple interactions in crystallographic structures, as listed in Table S7 of Supporting Information. The remaining 218 proteins interact with only one partner. A key example is proliferating cell nuclear antigen (PCNA), which interacts with 12 proteins and plays a vital role in DNA replication. However, its interactions vary across cancer types (in HNSC, PCNA engages with its full set of identified interactors, whereas in other cancers, its interaction network is more restricted). This structural perspective emphasizes the structural complexity of hub proteins and their potential as therapeutic targets. Other examples of hub proteins include HIF1A (with 5 interactors in HNSC) and CCT7, which shows a variable number of interactors across different cancer types (4 in LUSC, and 5 in both HNSC and COAD). Information regarding ligands of hub proteins is available in Table S8.

We identified a variable number and diversity of crystallographic hub proteins across different cancer types (Figure 7b), underscoring the distinct molecular landscapes that characterize each cancer subtype. Some cancers exhibit a higher prevalence of multi-interacting hub proteins, while others are dominated by proteins with fewer interactions. To provide deeper insights, we present a detailed comparison of the number of crystallographic hub proteins per cancer type, also distinguishing the number of interactors at their interfaces. This distinction captures the structural and functional characteristics of these hubs. Notably, some cancers, such as HNSC and LUSC, exhibit a higher number of hub proteins from a crystallographic perspective. This variation highlights the diverse roles of PPIs in driving oncogenesis across different cancers. From a structural perspective, 25 hub proteins also exhibit intrinsically disordered regions, which can interact with other proteins (Table S8), and whose interactions are often regulated through conformational changes that occur upon binding. Detailed information on crystallographic interactions, ligands, and disordered regions for two example hub proteins—VCP and HIF1A—is provided in Figure S4. In the case of the VCP protein, two interactions (VCP–VCP and VCP–FAF1) involve segments of disordered regions that are crystallized in the corresponding PDB entries (Figure S4a). Moreover, an allosteric-like pocket is bound to three ATP-like ligands (Figure S4b). In contrast, in the case of HIF1A, the disordered regions are not crystallized in the available PDB entries (Figure S4c), and therefore, it is not possible to assume that they are involved in the PPIs. However, even in this case, the allosteric-like pocket is found to be bound to two ligands (Figure S4d).

The diversity in hub protein behavior reflects the complexity of cancer biology. This observation suggests that future studies could explore potential correlations between these data and epidemiological trends or the aggressiveness of specific metastatic cancer subtypes [46].

**Figure 8:**
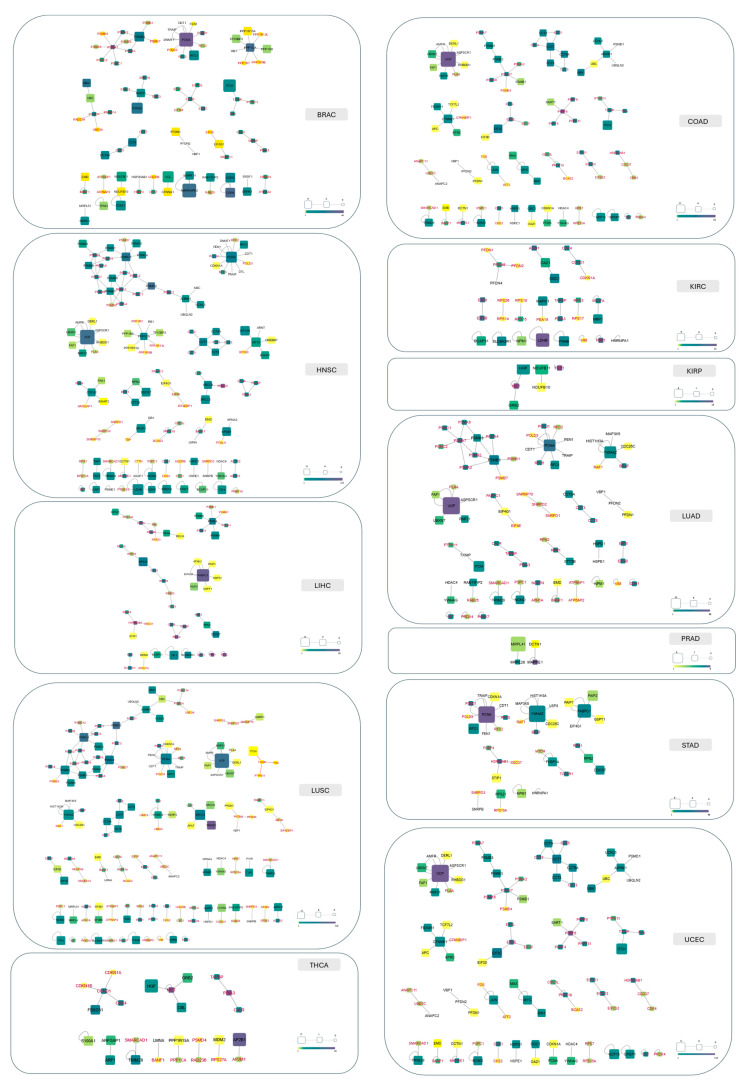
Network representations of 12 cancer types. In each network, nodes represent proteins and edges represent interactions. Node color reflects the total number of pockets (*interface* + *allosteric-like*): yellow indicates few pockets, and purple indicates many pockets. Node size represents the number of *interface* pockets, with larger nodes signifying a higher count of *interface* pockets. Node size is scaled within each individual network and is not comparable across networks. Nodes with red labels are proteins lacking *interface* pockets. Nodes without any shape indicate proteins with no pockets (neither *interface* nor *allosteric-like* pockets).

For each of the 12 cancer types, we constructed crystallographic network maps, where nodes represent proteins and edges denote interactions (Figure 8). Node color and size indicate the total number of pockets found on the protein and those specifically located at interaction interfaces, respectively. Notably, the same protein can behave differently across cancer types. Interaction maps vary significantly, from the sparser networks in PRAD and KIRP to the more intricate ones in HNSC and LUSC. This variation reflects (a) the availability of experimental data, (b) the number of crystallographic structures in the PDB, and (c) the presence of accessible pockets for small-molecule binding. Our method identifies buried regions with suitable physicochemical properties for ligand binding, making these maps an invaluable “navigation tool” for guiding drug discovery efforts.

#### Discovery of a New Ligandable Hub Pocket on VCP

**Figure 9:**
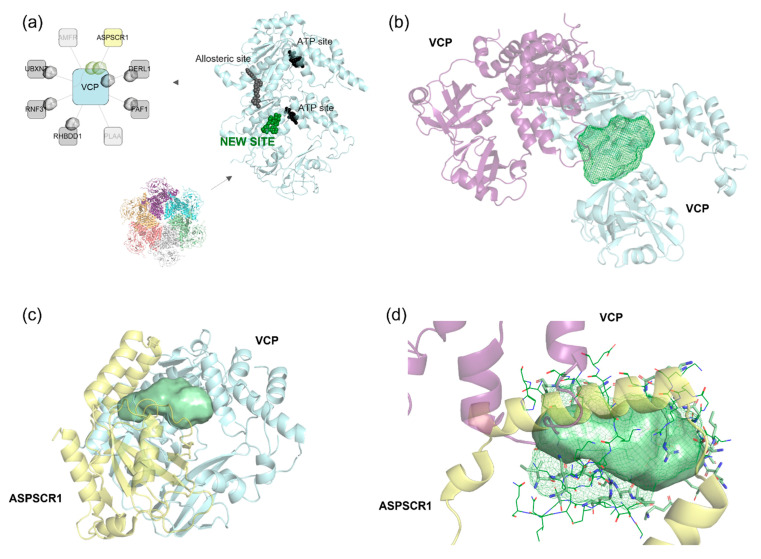
(**a**) The VCP interactors are represented in the VCP-centered network, highlighting partners that show at least one pocket at the interface. ASPSCR1 interactor is depicted in yellow (**left**). VCP hexamer is composed of different domains. One monomer is displayed in cyan cartoon. ATP binding sites and allosteric binding sites are represented in black and dark grey spheres, respectively. The new site identified in our analysis is represented in green spheres (**right**). (**b**) Interaction between the two monomers of VCP (PDB ID: 4ko8), shown in purple and cyan cartoon, respectively. A pocket identified at this interaction interface is illustrated as a dark green mesh. (**c**) Interaction between VCP and ASPSCR1 (PDB ID: 5ifs), depicted in green and yellow cartoon, respectively. The identified pocket on VCP interacting with ASPSCR1 is highlighted as a light green surface. (**d**) Overlap of the two identified pockets on two different VCP interactions: the pocket involved in homodimerization (represented as a dark green mesh) and the pocket mediating interaction between the monomer and ASPSCR1 (depicted as a light green surface). Residues involved in these interactions are highlighted in stick and line representation.

Valosin-containing protein (VCP), a key hub in multiple cancers (COAD, HNSC, LUAD, LUSC, UCEC), regulates the ubiquitin-proteasome system and autophagy. It interacts with ~40 known co-factors, directing its activity toward different substrates. Our study identified five crystallographically resolved VCP co-factor interactions (ASPSCR1, DERL1, FAF1, RHBDD1, UBXN7) with available pocket interfaces (Figure 9a). These interactions are overexpressed in all five cancers, except for VCP-DERL1 in LUAD. Not all VCP-mediated interactions are equally characterized. For example, no pockets were detected in the VCP-AMFR complex, limiting potential drug targets (Figure 9a). In contrast, pockets were identified on both partners in the VCP-DERL1 complex, opening avenues for targeted drug design. This variable pocket presence across interactions highlights the therapeutic potential of different sites within the VCP network.

Existing VCP inhibitors include ATP-competitive and allosteric molecules [47]. Our study identified a previously unreported binding site located on the N-terminal domain, distinct from ATP or allosteric binding sites (Figure 9a). This site was observed in both the VCP hexamer (Figure 9b, dimer shown for clarity) and in the VCP-ASPSCR1 interaction (Figure 9c). Notably, ASPSCR1 binding disrupts the VCP hexamer, forming a heterotetramer that inhibits VCP ATPase activity [48]. The newly identified site comprises two key overlapping pockets: one involved in hexamer formation (Figure 9b) and another engaged in the VCP-ASPSCR1 interaction (Figure 9c). Their spatial alignment (Figure 9d) suggests that ASPSCR1 competes with VCP itself, specifically through its α-helical domain, which is essential for hexamer dissociation. This spatial overlap supports the idea that this region could be a prime target for developing new VCP modulators, potentially disrupting or regulating dimerization-related functions. Interestingly, VCP adopts different conformations depending on the interacting partner, leading to distinct pocket shapes (Figure 9d). This structural flexibility is crucial for drug design, as it enables selective engagement of interaction-specific residues.

Furthermore, the pocket involved in the VCP-DERL1 interaction is located in a completely different region from the newly identified ASPSCR1-related pocket (see Figure S3, Supporting Information). This underscores the functional and structural versatility of hub proteins, which do not always rely on a single region for interacting with different partners. In conclusion, by integrating 3D structural insights with network analysis, our study highlights VCP as a highly connected hub with multiple ligandable pockets. The newly identified site offers an exciting target for next-generation VCP inhibitors, paving the way for innovative strategies in cancer therapeutics.

In some cases, however, promiscuous pockets are observed, as in the VCP example, where the same binding site may accommodate different ligands or interactors. This variability suggests that certain regions of these hubs might offer a broader scope for drug design, potentially enabling the development of molecules that can target multiple interactions or modulate diverse biological processes simultaneously.

The concept of hub protein promiscuity has been critically examined in recent research [49]. The authors argue that what appears as promiscuity is often a misinterpretation. It is acknowledged that a single gene is frequently translated into several isoform proteins and that post-translational modifications can take place. It was argued that the different protein variants can be grouped into a single node in the interaction maps; this generates the illusion of a single protein engaging in numerous interactions. In our study, we address this issue by analyzing crystallographic structures of specific oncoPPIs and, above all, by focusing on specific pockets involved in interactions, providing a more accurate and detailed perspective on whether a given pocket is promiscuous or highly specific.

## 3. Materials and Methods

### 3.1. Protein Validation Set

A validation set was employed to assess the capability of the BioGPS algorithm in correctly predicting known binding sites, as summarized in Table S1 of the Supporting Information. A set of 15 PPI complexes related to cancer was obtained by extracting data from the 2P2DIdb database, whereas a collection of 7 PROTAC POI structures was manually selected by inspecting the PDB and consulting the literature [28,50]. The systems preparation and pockets detection workflow are reported in the next section. The MCC metric was calculated to compare the crystallographic residues with those sampled by BioGPS, as previously reported [24]. A value of MCC of +1 indicates an ideal prediction; a value of MCC of 0 indicates predictions no better than random; and a value of MCC of −1 indicates a complete disagreement between predicted and observed cases. All statistics are reported in Table S1 of the Supporting Information.

### 3.2. oncoPPI Dataset

Gene names of activated oncoPPIs were collected from the supplementary material of the study by Gulfidan et al. [8]. The gene names were mapped to UniProt identifiers using the UniProt web service [51]. The resulting UniProt protein name pairs were matched with Interactome3D [52] entries downloaded on 5 February 2024. Specifically, a curated, non-redundant version of the Interactome 3D database was used, where only one representative PDB structure is retained for each protein complex, ensuring a streamlined evaluation of structural data. Once the PDB codes for the complexes were identified, the corresponding structural files were downloaded directly from the PDB.

### 3.3. Protein Preparation

All the target proteins were first processed by employing the BioGPS tool (v. 24.01.5) to prepare the input protein structures for further analysis. All nucleic acids, ligands, crystallographic artifacts, and water molecules were removed, while the cofactors (e.g., NAD, FAD, GSH) were retained. Notably, only the two chains participating in the 3D oncoPPIs were considered.

### 3.4. Pockets Detection

All the possible protein pockets were sampled by using BioGPS v. 24.01.5 [27], developed and licensed by Molecular Discovery Ltd. In summary, the protein structure is positioned within a three-dimensional grid, where the level of burial at each point is calculated, factoring in hydrophobicity using the GRID DRY probe. Points that are sufficiently buried and hydrophobic are grouped together, after which an erosion/dilation algorithm is applied to smooth the identified pocket regions. Only those pockets that are large enough to accommodate drug-like molecules are retained for further analysis.

1. In the validation set, for each protein, the pockets were calculated: (i) on the inhibitor-bound form, (ii) on the protein-bound form, or (iii) on the PROTAC-bound form. In all cases of complexes with inhibitors, protein partners, or PROTACs, any binder was previously extracted to find the cavity involved in the binding.
2. In the 3D oncoPPI datasets, the pockets were calculated on each individual partner separately, by splitting the crystallographic complex (i.e., detached partner) and by considering the entire complex (i.e., complexed partner). In the first case, each individual chain representing the partner protein was used as input for BioGPS to collect its pockets. In the second case, the original crystallographic complex of the two chains was used as input for pocket detection.

### 3.5. Classification of Pockets in the 3D oncoPPI Dataset

The pockets identified in the oncoPPIs dataset were categorized into three distinct categories based on their location and involvement in PPIs:

1. *Interface* pockets: these pockets were identified on individual (detached) protein partners and are located in regions involved in PPIs. A pocket is classified as an *interface* pocket if the fraction of volume of the interacting partner contained within the pocket is greater than 0.
2. *Allosteric-like* pockets: these pockets were identified on individual (detached) protein partners that do not correspond to any regions directly involved in PPIs.
3. *Equilibrium* pockets: these pockets were calculated by considering the whole crystallographic complex and are located in regions that define the interaction interface between the two protein partners. Specifically, for a pocket to be classified as an equilibrium pocket, it must be composed of residues contributed by both interacting partners.

### 3.6. Ligand-Bound Pockets

This step aimed to identify pockets belonging to the three mentioned categories (i.e., *interface*, *allosteric-like*, and *equilibrium*) containing pre-existing co-crystallized ligands in PDB structures. For each partner protein in the 3D oncoPPI dataset, all PDB structures corresponding to the same UniProt code were identified and downloaded. These structures were then aligned using an in-house script based on PyMOL v2.5 [53]. Protein alignments with an RMSD lower than 3.0 Å were excluded. Following alignment, all potential ligands were extracted using BioGPS, excluding cofactors, metals, and crystallization artifacts. As a result, ligands were aligned to the reference structures of each protein in the 3D oncoPPI dataset. Subsequently, for each protein, the overlap between the volume of aligned ligands and its identified pockets (i.e., *interface*, *allosteric-like*, and *equilibrium*) was calculated. A pocket was defined as a “ligand-bound pocket” if this ratio exceeded 0.3, indicating that at least 30% of the atomic structure of the aligned ligand resided within the pocket.

### 3.7. Ligands’ Physicochemical Properties

The ligand collection was initially profiled using VolSurf+ descriptors (v1.1.2) consisting of a set of 128 molecular descriptors from 3D MIFs, which are particularly relevant to ADME prediction and are also easy to interpret [20]. The generated multidimensional descriptor space was then simplified by applying principal component analysis (PCA). This reduction step facilitated the visualization and interpretation of the data while retaining the most relevant features. All the statistical and descriptive analyses were carried out using Rstudio v. 4.0.3.

### 3.8. Ligands’ Classification

The collected ligands were categorized based on their clinical phase and, where available, their therapeutic classification. All ligands were initially searched in DrugBank [41]. A total of 150 ligands were identified with a DrugBank code and classified into three main categories: *approved*, *clinical*, and *preclinical*. For ligands classified as *approved*, their ATC codes were also recorded. For ligands classified as *clinical*, their potential therapeutic and clinical trials were noted based on DrugBank web server information if available (DrugBank group = Investigational). On the other hand, *preclinical* ligands are compounds belonging to the DrugBank group flagged as experimental or nutraceutical. A total number of 259 ligands, not found in DrugBank, were classified as *clinical* or *preclinical* based on annotations available in ChEMBL v.35 [42]. A total of 258 ligands were categorized as *no_data* because they were not annotated in either the DrugBank or ChEMBL databases. For these compounds, the PubChem ID was recorded if available.

### 3.9. 3D oncoPPI Network

All PPIs were categorized based on their association with specific types of cancer. For each protein, two key metrics were recorded: the number of *interface* pockets and the number of *allosteric-like* pockets. These values were used as input for a network analysis performed with Cytoscape (version 3.8.0) [54]. Within the network analysis, a protein was defined as a *hub* if it was associated with two or more interacting partners, emphasizing its centrality and potential importance in the interaction network.

### 3.10. Pockets Physicochemical Properties

Each pocket was analyzed using GRID probes (H, DRY, OH2, O, and N1) to assess various types of interactions [26]. Specifically, the H probe was used for shape evaluation, the DRY probe was used to identify hydrophobic interactions, the OH1 probe was used to identify hydrophilic interactions, the O probe was used for hydrogen bond acceptor sites, and the N1 probe was used for hydrogen bond donor interactions. A set of geometrical and energetic descriptors was computed for the pocket collection using BioGPS software v. 24.01.5 [27], providing easily interpretable results.

▪ Globularity: quantifies the degree of sphericity of the pocket. It is equal to 1.0 for perfect spherical objects, whereas it assumes values lower than 1.0 for real spheroidal ones;
▪ Rugosity: indicates the presence of molecular wrinkles or creases on the pocket surface expressed as the ratio of volume to surface. The higher the ratio, the higher the rugosity;
▪ Hydrophobic volume: proportional to the number of points in the DRY field, filtered to include only those with energy lower than −0.5 Kcal/mol;
▪ Hydrophilic volume: proportional to the total number of points in the OH2 field, considering only points with energy below −3.5 Kcal/mol;
▪ Exposed to solvent: describes the surface of the pocket accessible to the solvent and not in contact with protein residues. It is proportional to the external points in the H field that are at least 2.2 Å away from the protein atoms;
▪ Buried volume: measures the volume of points embedded within the protein cavity, calculated by summing the “collisions” of 50 vectors intersecting with the protein surface. Each “collision” adds to the buriedness, and the final value is an average of all the values across all points. The reported value refers to the pocket points with low buried volume.

All the statistical and descriptive analyses were carried out using Rstudio v2024.09.1 [55].

## 4. Conclusions

This study investigates 3D protein–protein interactions (3D oncoPPIs) involved in various cancers, focusing on protein pockets as critical regulatory sites. These pockets are promising targets for modulating protein functions and guiding cancer therapy. To support this, we developed a detailed database of essential protein regions linked to cancer phenotypes, aiding drug design efforts.

Two main analyses were conducted: (a) identification of interface pockets essential for modulator design, and (b) detection of surface pockets suitable for PROTAC-mediated degradation. The in silico predictions showed 75% accuracy for known inhibitor sites and 100% for PROTAC targets. Mapping 314 3D oncoPPIs revealed three pocket types: 248 *interface*, 2552 *allosteric-like*, and 812 *equilibrium* pockets.

Ligand-binding analysis showed that 11.29% of *interface*, 7.13% of *allosteric-like*, and 11.94% of *equilibrium* pockets are ligand-bound, providing a strong basis for drug development. Ligands were classified by ATC code and clinical phase, highlighting therapeutic potential. STAD, KIRC, and UCEC had the most ligand-bound interface pockets, suggesting high suitability for targeted therapies. Clinical case studies on proteins like S100A1, HIF1α, MTOR, NRP1, and CTNNB1 underscore the clinical relevance of these findings.

Physicochemical comparisons between bound and unbound pockets indicate a broader ligandable space than currently explored. Examples like PCNA and BRAF suggest untapped drug potential. Moreover, structural similarities across unrelated proteins, detected via 3D molecular interaction fields (MIFs), further expand the targeting landscape. Finally, analysis of hub proteins, such as VCP, revealed their central role in cancer PPI networks and potential as drug targets.

This study introduces the first comprehensive reference of protein and pocket structures in 3D oncoPPIs, freely available to the scientific community for drug discovery research at https://github.com/moldiscovery/OncoPPI-pocketome.

Future developments of this work will include an analysis of the impact of mutations on both the shape and interactions of the pockets, as well as the inclusion of additional cancer types, including hematologic malignancies such as leukemia, multiple myeloma, and lymphoma. Finally, a three-dimensional analysis of intrinsically disordered regions may offer further insights and avenues for development.

In conclusion, the insights gained from this analysis extend beyond cancer, positioning our approach as a versatile and adaptable resource for prioritizing high-value therapeutic candidates across a broad range of diseases.

## Figures and Tables

**Table 1:** Top five occupied ligand pockets for each category (*interface*, *allosteric-like*, and *equilibrium*).

**Interface pockets**		
**n° X-ray ligands**	**Liganded partner protein**	**Interacting partner protein**
16	Transforming protein RhoA	Rho GTPase-activating protein 1
11	14-3-3 protein zeta/delta	Mitogen-activated protein kinase kinase kinase 5
9	Proteasome subunit beta type-6	Proteasome subunit beta type-4
6	Neuropilin-1	Vascular endothelial growth factor A
4	Proliferating cell nuclear antigen	DNA polymerase delta subunit 3
**Allosteric-like pockets**		
**n° X-ray ligands**	**Liganded partner protein**	
177	Mitogen-activated protein kinase 14	-
126	Mitogen-activated protein kinase 1	-
60	Serine/threonine-protein kinase B-raf	-
43	Fructose-1,6-bisphosphatase 1	-
35	Peptidyl-prolyl cis-trans isomerase FKBP1A	-
**Equilibrium pockets**		
**n° X-ray ligands**	**Liganded partner protein 1**	**Liganded partner protein 2**
27	Transforming protein RhoA	Rho guanine nucleotide exchange factor 12
23	Serine/threonine-protein kinase PAK 4	Cell division control protein 42 homolog
22	Proteasome subunit beta type-1	Proteasome subunit beta type-8
10	Transforming protein RhoA	Rho GTPase-activating protein 1
10	Proteasome subunit beta type-4	Proteasome subunit beta type-6

**Table 2:** Summary of computed MIF descriptors.

Pocket	Rugosity	Globularity	Hydrophilic Volume	Hydrophobic Volume	Buried Volume	Exposition to Solvent
**Ligand-bound interface PCNA**	2.213	0.820	52.312	46.406	645.559	159.188
**Allosteric-like BRAF**	2.159	0.851	52.734	32.062	552.502	187.312

## Data Availability

Files of proteins and pockets can be downloaded from https://github.com/moldiscovery/OncoPPI-pocketome. The BioGPS (v. 24.01.5) and Volsurf+ (v1.1.2) software, containing executables for pocket detection and descriptors computation, are available from https://www.moldiscovery.com, and trial licenses are available to both commercial and academic users.

## Supplementary Materials

The following supporting information can be downloaded at: https://www.mdpi.com/article/10.3390/ph18070958/s1. Figure S1: Structural and quantitative overview of protein dimers, showing the distribution of *interface* and *allosteric-like* pockets in homodimers and heterodimers; Figure S2: Density plots comparing physicochemical descriptors of all detected pockets versus ligand-bound pockets across three pocket types; Figure S3: VCP hub protein and its interactors, with a focus on the VCP–DERL1 interaction; Figure S4: Detailed information on crystallographic interactions, ligands, and disordered regions for two example hub proteins.; Table S1: Validation dataset; Table S2: Numbers of mapped protein–protein interactions (both activated and repressed); Table S3: Numbers of the detected pockets, classified into the three categories for each cancer type; Table S4: Percentages of residues from both partners that define an *equilibrium* pocket; Table S5: All ligands identified within the detected pockets; Table S6: Comprehensive information on ligands (clinical and therapeutic classification); Table S7: List of hub proteins; Table S8: Detailed information of ligands and disordered regions of hub proteins
