# Supplementary material for: Target Mapping in Cancer: Ligandable Protein Pockets on 3D OncoPPI Networks
Source: Pharmaceuticals (Basel). 2025 Jun 25;18(7):958. doi: 10.3390/ph18070958 (PMC12298929; doi:10.3390/ph18070958)
Supplement: Supplementary file 1 [file pharmaceuticals-18-00958-s001.zip › pharmaceuticals-3673114-supplementary.pdf]

SUPPORTING INFORMATION

Targeted shooting in cancer: ligandable protein pockets on 3D oncoPPI networks

Daniela Trisciuzzi[1], Orazio Nicolotti[1], Gabriele Cruciani[2], Gabriele Menna[3], Lydia Siragusa[3,4,*]

1. Department of Pharmacy, Pharmaceutical Sciences, Università Degli Studi di Bari "Aldo Moro", Bari, Italy

2. Laboratory for Chemoinformatics and Molecular Modelling, Department of Chemistry, Biology and Biotechnology, University of Perugia, Perugia, Italy.

3. Molecular Discovery, Kinetic Business Centre, Theobald Street, Elstree, Borehamwood, Hertfordshire WD6 4PJ, UK.

4. Molecular Horizon srl, via Montelino 30, 06084 Bettona (PG), Italy

* Corresponding author: lydia@moldiscovery.com

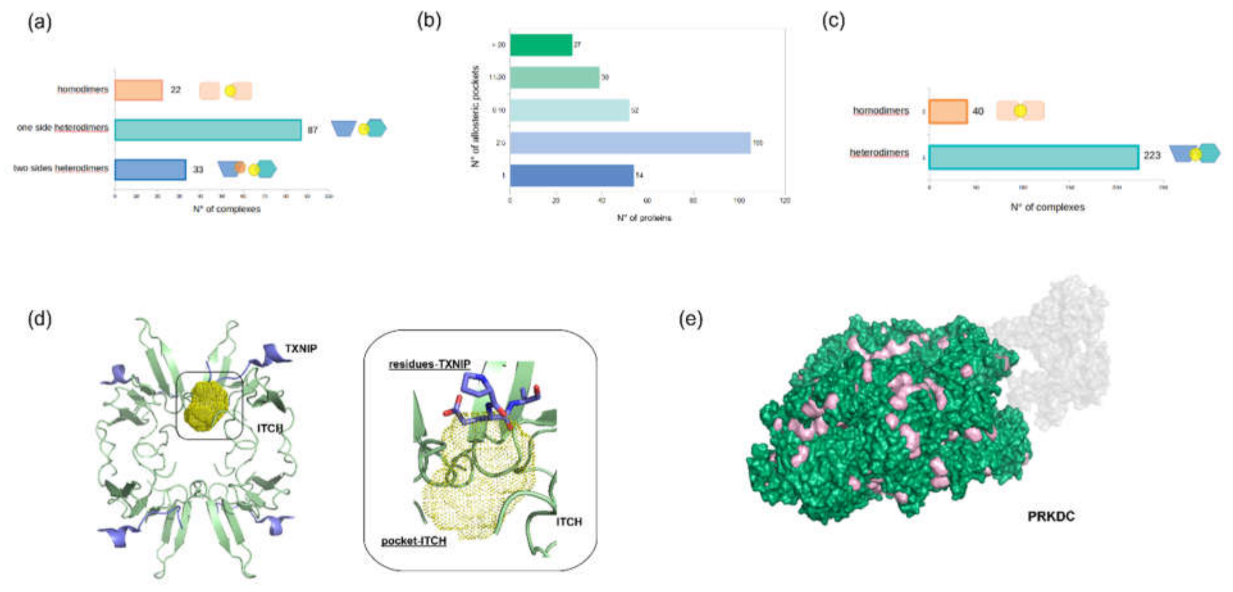

Figure S1. (a) Histogram showing the number of homodimers and heterodimers categorised by the presence of interface pockets on one or both partners; (b) (a) Distribution of the number of proteins with different ranges of allosteric pockets. (c) Histogram showing the number of homodimers and heterodimers categorised by the presence of equilibrium pockets; (d) the ITCH-TXNIP heterodimer. ITCH and TXNIP are shown in cyan and blue cartoon, respectively with an enlarged region showing a pocket on ITCH containing TXNIP residues (blue sticks) (PDB ID: 5cq2); (e)Protein with their respective allosteric pockets displayed in pink surface. The interacting partners are represented in light grey (PDB IDs: 7k0y).

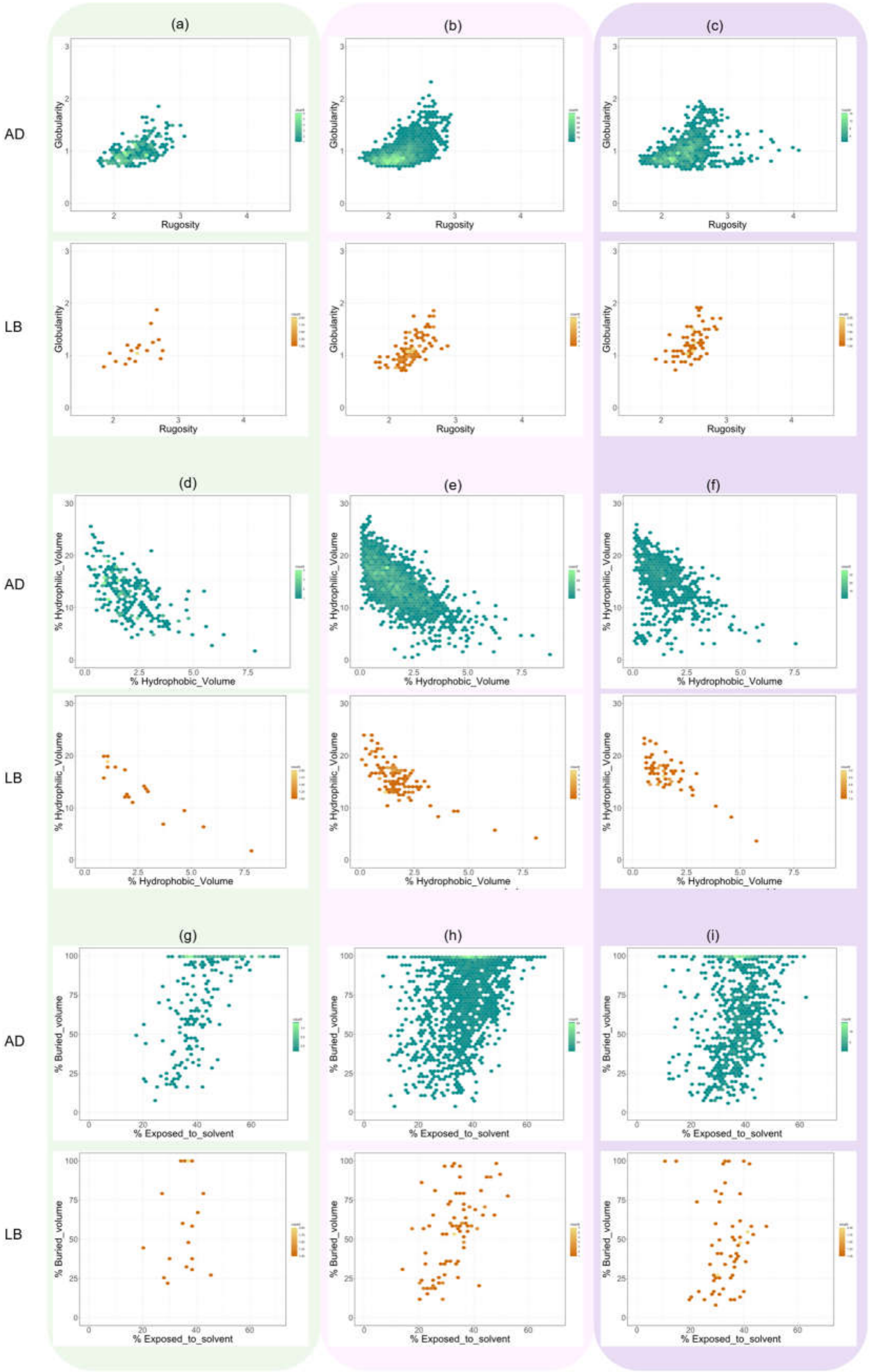

Figure S2. Density plots reporting a comparison of the calculated physicochemical descriptors for all detected pockets (AD, green gradient) and the ligand-bound pockets (LB, orange gradient). The shape and the peak represent the population of the paired descriptors.

Interface pockets (green left hand-side panel), allosteric-like pockets (pink middle panel), equilibrium pockets (violet right panel). (a, b, c) Globality vs Rugosity. (d, e, f) Hydrophilic vs hydrophobic volume. (g, h, i) Burieded volume *vs* exposition to solvent.

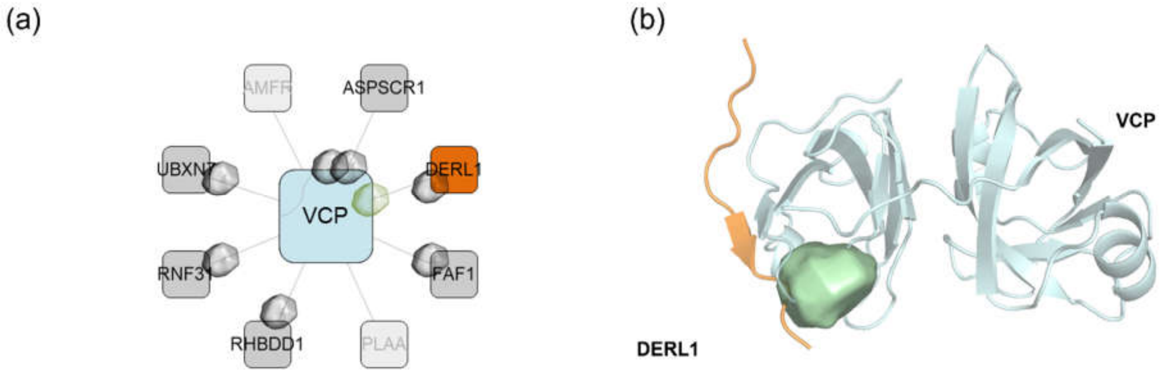

Figure S3. a) VCP hub protein and its related interactors. b) Interaction between VCP and DERL1 depicted in cyan and orange cartoon, respectively. The identified pocket on VCP interacting with DERL1 is highlighted as a light green surface.

a)

	PDB entry	Liganded pocket	Ligand(s)	Disordered regions ['1-20', '462-472']
VCP-VCP	4ko8	/	/	Partially solved (‘14-20’), interacting
VCP-ASPSCR1	5ifs	Pocket_003_5ifs (allosteric-like)	ADP, AGS,ATP	Not solved
VCP-DERL1	5glf	/	/	Not solved
VCP-FAF1	3qq8	/	/	Partially solved (‘10-20’), not interacting
VCP-RHBDD1	5epp	/	/	Not solved
VCP-UBXN7	5x4l	/	/	Not solved
VCP-AMFR	3tiw	/	/	Not solved
VCP-PLAA	3ebb	/	/	Not solved
VCP-RNF3	4p0a	/	/	Not solved

b)

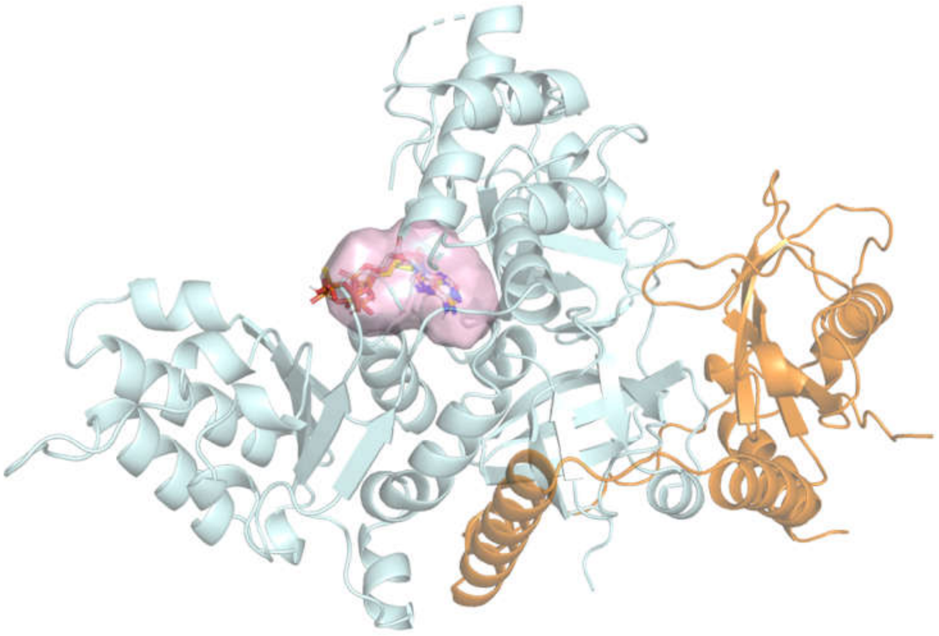

c)

	PDB entry	Liganded pocket	Ligand(s)	Disordered regions ['403-603', '530-698', '776-826', '791-804', '815-824']
HIF1A-ARNT	4h6j	/	/	Not solved
HIF1A-EP300	1l3e	/	/	Not solved
HIF1A-CREBBP	1l8c	/	/	Not solved
HIF1A-EGLN1	5l9v	Pocket_001_5l9v (allosteric-like)	OGA, AKG	Not solved
HIF1A-HIF1AN	5jwp	/	/	Not solved

d)

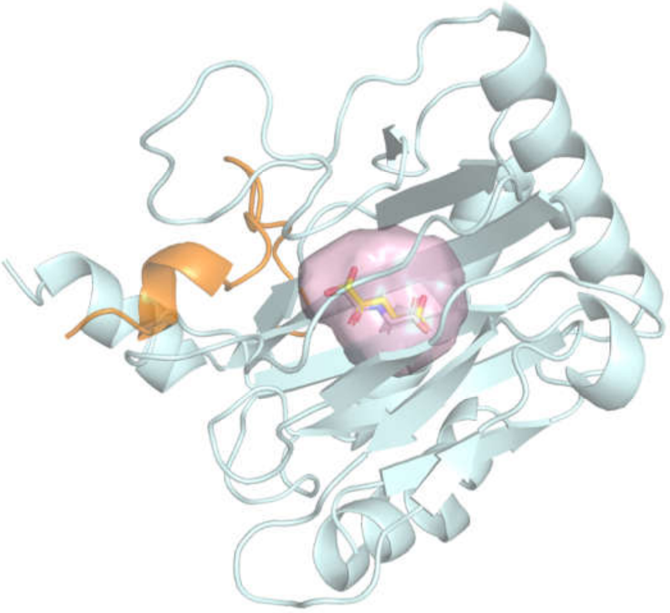

Figure S4. Detailed information on crystallographic interactions, ligands, and disordered regions for two example hub proteins. a,c) The tables report the crystallographic structure of the PPI, the liganded pocket, the detected ligands (coming from aligned PDB entries, as listed in Table S5), and the disordered regions, indicating whether they are crystallized and, if so, whether they participate in the corresponding PPI. b) VCP–ASPSCR1 and d) HIF1A–EGLN1 interactions with its liganded pockets. Ligands are displayed as colored sticks whereas liganded pocket are shown as a pink surface.
